# Evaluating the impact of fast-fMRI on dynamic functional connectivity in an event-based paradigm

**DOI:** 10.1371/journal.pone.0190480

**Published:** 2018-01-22

**Authors:** Ashish Kaul Sahib, Michael Erb, Justus Marquetand, Pascal Martin, Adham Elshahabi, Silke Klamer, Serge Vulliemoz, Klaus Scheffler, Thomas Ethofer, Niels K. Focke

**Affiliations:** 1 Werner Reichardt Centre for Integrative Neuroscience, Tuebingen, Germany; 2 Department of Biomedical Magnetic Resonance, University Hospital Tuebingen, Tuebingen, Germany; 3 Department of Neurology/Epileptology, University Hospital Tuebingen and Hertie Institute of Clinical Brain Research, Tuebingen, Germany; 4 Graduate School of Neural and Behavioural Sciences/International Max Planck Research School, University of Tuebingen, Tuebingen, Germany; 5 MEG-Center, University of Tuebingen, Tuebingen, Germany; 6 Department of Neurology, University Hospital of Geneva, Geneva, Switzerland; 7 Max-Planck-Institute for Biological Cybernetics, Tuebingen, Germany; 8 Clinical Neurophysiology, University Medicine, Goettingen, Germany; University of Cambridge, UNITED KINGDOM

## Abstract

The human brain is known to contain several functional networks that interact dynamically. Therefore, it is desirable to analyze the temporal features of these networks by dynamic functional connectivity (dFC). A sliding window approach was used in an event-related fMRI (visual stimulation using checkerboards) to assess the impact of repetition time (TR) and window size on the temporal features of BOLD dFC. In addition, we also examined the spatial distribution of dFC and tested the feasibility of this approach for the analysis of interictal epileptiforme discharges. 15 healthy controls (visual stimulation paradigm) and three patients with epilepsy (EEG-fMRI) were measured with EPI-fMRI. We calculated the functional connectivity degree (FCD) by determining the total number of connections of a given voxel above a predefined threshold based on Pearson correlation. FCD could capture hemodynamic changes relative to stimulus onset in controls. A significant effect of TR and window size was observed on FCD estimates. At a conventional TR of 2.6 s, FCD values were marginal compared to FCD values using sub-seconds TRs achievable with multiband (MB) fMRI. Concerning window sizes, a specific maximum of FCD values (inverted u-shape behavior) was found for each TR, indicating a limit to the possible gain in FCD for increasing window size. In patients, a dynamic FCD change was found relative to the onset of epileptiform EEG patterns, which was compatible with their clinical semiology. Our findings indicate that dynamic FCD transients are better detectable with sub-second TR than conventional TR. This approach was capable of capturing neuronal connectivity across various regions of the brain, indicating a potential to study the temporal characteristics of interictal epileptiform discharges and seizures in epilepsy patients or other brain diseases with brief events.

## Introduction

Identifying and understanding brain connectivity from non-invasive and *in-vivo* functional magnetic resonance imaging (fMRI) is desirable both for physiological and pathological processes. However, previous investigations on functional connectivity (FC), especially in resting state studies, have relied largely on static approaches, i.e. correlation of signals calculated for the entire scan duration [1]. Thus, these approaches represent the functional relationship between regions without reflecting the dynamics of functional connectivity over time. Recent studies have shown that FC using fMRI during resting state are not sustained, but vary with time [2–7]. One of the most commonly used strategies for examining such dynamics in FC is the sliding window approach, i.e. a repeated application of a (static) FC metric on a gradually changing time period [2, 5]. One of the most important parameters of this approach is the choice of the employed window size: ideally, the window should be large enough to permit robust estimation of FC and yet be small enough to detect potential transients [8, 9]. Given the temporal resolution of conventional fMRI (2–3 s), possible window sizes are limited. Thus, most dynamic FC (dFC) studies done in resting-state experiments were based on the slow fluctuations between 0.01 and 0.1 Hz [10, 11]. However, dFC analysis of brief events could be biologically very relevant. One such condition is epilepsy that is characterized by spontaneous interictal epileptiform discharges (IED, i.e. spikes or sharp waves). These IED, recorded by electroencephalography (EEG) or magnetoencephalography, occur in many patients with epilepsy and are regarded as the electrophysiological signature of epileptic activity. IED are brief events in the order of 100 ms or burst of up to a few seconds and their localization is a good surrogate for localizing the epileptogenic zone, i.e. the source of seizures in the individual patients. Such IEDs can be captured and localized with combined EEG-fMRI. Several studies have successfully used event-related designs to assess IED-associated fMRI activity changes (for a review see [12]). However, methods to capture the rapid temporal dynamics of these brief transients have been seldom explored. Static FC in fMRI has also been recently used to investigate brain networks in epilepsy [13, 14]. Enabling dFC analysis of IED in individual patients could, thus, be clinically very relevant.

Recent advances in imaging using multiband (MB) excitation have reduced the time to acquire fMRI data of the whole brain [15]. Using MB radio frequency pulses with slice-selective gradients, multiple brain slices can be acquired during a single echo planar imaging (EPI) acquisition without sacrificing spatial resolution. The term MB is used to describe slice acceleration factors created by the radio frequency (RF) pulses. A higher temporal resolution and, thus, an increased number of data points, offers several advantages including improved statistical power [16], a better analysis of the onset and the shape of the hemodynamic response, and can enable a better correction of physiological noise (for a review, see [15]).

A higher temporal resolution increases the number samples per window, which in turn could provide a more robust estimation of FC, therefore, we first investigated the impact of repetition time (TR) on blood-oxygen-level-dependent (BOLD) fMRI derived dFC in a sliding window approach in an event-related, visual-stimulation paradigm. Secondly, we investigated the effect of various windows sizes on dFC estimates. Thirdly, we examined the spatial distribution of dFC across the brain. Finally, we tested the feasibility of the optimized dFC method in three epilepsy patients with different epilepsy syndromes and tested the plausibility of the results in relation to the clinical information.

## Methods

### Participants and study design

To characterize the impact of technical parameters (window size, TR), we considered the data from our previous study [17]. Fifteen healthy volunteers (10 male, mean age 24.4 ± 2.0) participated in the study (“control task”). This study was conducted in accordance with the Helsinki convention and approved by the local Ethics Committee of the Medical Faculty of the University Tuebingen. First, a short block paradigm (duration: 3 min) with blocks of flickering checkerboards and blocks with a fixation cross (duration of each block: 20 s) was used to derive a functionally-defined region of interest (ROI) within the visual cortex. Then, an event-related design (duration of paradigm: 10 min) with brief, pseudo-random checkerboard stimulation periods (duration: 500 ms) was applied. To ensure that the participants remained vigilant, they were instructed to focus on the fixation cross at the center of the screen and press the response button with their right thumb whenever the visual stimulus was presented.

In addition, three patients (Table 1) were recruited in the Neurology/Epileptology department of the University Hospital Tübingen (Germany). Written informed consent was obtained prior to the measurement, in accordance with the ethics committee guidelines. A resting state (eyes closed) paradigm was employed for 30 min (EEG-fMRI). As subject motion can severely influence the FCD estimation [18], we checked that all included subjects and patients exhibited little head motion (< 1.5 mm) in our study.

**Table 1:** Clinical data of epilepsy patients.

Patient	Age	Diagnosis	Clinical details / Hypothesis	IEDs/ 30 min	Medication (daily dose in mg)
Case 1	30 years	focal epilepsy, lesional	*MRI*: low-grade tumor right mesio parieto-occipital	20	levetiracetame (1000), lamotrigine (300), lacosamide (200)
			*EEG*: right temporal spikes with shifting maxima		
			**Hypothesis/Syndrome**: right mesio parieto-occipital		
Case 2	21 years	generalized epilepsy	*MRI*: normal	3	none
			*EEG*: gen. 3Hz-spike waves		
			**Hypothesis/Syndrome**: childhood absence epilepsy		
Case 3	57 years	generalized epilepsy	*MRI*: normal, except some microvascular lesions	20	lamotrigine (400)
			*EEG*: gen. 3Hz spike waves		
			**Hypothesis/ Syndrome**: juvenile absence epilepsy		

### Data acquisition

Structural T1-weighted images (repetition time (TR) = 2300 ms, echo time (TE) = 3.03 ms, inversion time (TI) = 1100 ms, voxel size: 1 × 1 × 1 mm^3^) and functional gradient echo EPI images (40 axial slices acquired in right-left phase encoding direction, slice thickness 3 mm without gap, TE = 30 ms, voxel size: 3 × 3 × 3 mm^3^) were acquired with a 3 tesla (T) scanner (Siemens TIM TRIO, Erlangen, Germany). For the control task, MB accelerated EPI images were acquired at MB-1 (TR = 2.64 s, flip angle (FA) = 90°), MB-2 (TR = 1.32 s, FA = 65°), MB-4 (TR = 0.66 s, FA = 50°) and MB-8 (TR = 0.33 s, FA = 37°). For offline distortion correction, we acquired EPI images at MB-1 in opposite phase encoding directions. We used the MB accelerated EPI sequences developed at the Center for Magnetic Resonance Research (CMRR, University of Minnesota) for the Human Connectome Project (15). Heart rate and respiration frequency were acquired with the pulse oximeter and respiratory belt (standard MR compatible equipment supplied with the 3T scanner, Siemens, Erlangen, Germany).

The sequence parameters for the structural T1-weighted and fMRI data acquisition were identical for the patient data acquisition as described above. Hd-EEG-fMRI was recorded using a 256-channel EEG system (Electrical Geodesics, Inc., Eugene, OR, U.S.A) in a 3T scanner (Siemens MAGNETOM Prisma, Siemens AG, Erlangen, Germany). EEG and fMRI were acquired simultaneously and continuously for 30 minutes at MB-4 (TR = 0.66 s, flip angle (FA) = 50°). Physiological and image data acquisition for distortion correction was similar to that of control task. During the recording, the internal ventilation system and the helium pump of the MR scanner were switched off to reduce contamination of the EEG by external artifacts [19].

### Data analysis

#### Preprocessing (controls and patients)

FSL topup (http://www.fmrib.ox.ac.uk/fsl) was used to estimate a fieldmap, which was applied to all functional images for EPI distortion correction. Then, images were preprocessed using statistical parametric mapping software (SPM8, Wellcome Trust Center for Neuroimaging, London, UK). Preprocessing comprised realignment, correction of timing difference in slice acquisition (the first acquired slice after half of the volume acquisition was taken as reference, e.g. the 21st slice for a volume of 40 slices) and normalization into MNI space (Montreal Neurological Institute, voxel size: 3 × 3 × 3 mm^3^). To remove spurious sources of variance, the fMRI time series were preprocessed as follows: six head motion parameters, averaged signals from CSF and white matter, global signal regression (GSR) of fMRI signal and eight physiological noise regressors (sine and cosine of phase of heart beat and respiration up to the second order, respectively) as calculated by RETROICOR [20] were removed by partial regression using the REST toolbox [21]. High-resolution T1-weighted images were used for cortical reconstruction and volumetric segmentation using the Freesurfer 5.3 software package (https://surfer.nmr.mgh.harvard.edu/fswiki). In brief, the processing contains intensity inhomogeneity correction, removal of non-brain tissue, intensity normalization, tissue segmentation [22], automated topology correction, surface deformation to generate the gray/white matter boundary, and parcellation of the cerebral cortex [23]. The atlas generated by Freesurfer (Desikan-Killiany) was normalized into MNI space to the same voxel resolution of fMRI data (using nearest neighbor interpolation). In the same way the 7-network resting-state atlas [24], that is based on functional parcellation to the voxel resolution of our normalized fMRI data, was also brought into the same space.

Gradient artifacts from the EEG data were removed using a moving averaged template subtraction method, followed by artifact residual removal using optimal basis set and adaptive noise cancellation [25, 26] using EGI Netstation version 5 based on previous work [27]. Onset and end time of IED were marked and classified by an experienced neurologist, according to both spatial distribution and morphology.

#### Functional MRI analysis

First, we defined a ROI based on a second-level random effects analysis of the activation during the visual block paradigm (checkerboard > fixation cross) based on a height threshold of p < 0.05 (family-wise-error (FWE) corrected, corresponding to a t > 7.63). The hemodynamic response was extracted from the ROI (for a time window of 20 seconds after stimulus onset) and averaged across events and subjects, separately for all TRs.

#### Dynamic FCD analysis in the visual control task

Voxels within the grey matter as determined by subject-specific mask based on the SPM segmentation (probability threshold = 0.2) were analyzed using the DynamicBC toolbox [28] to assess the functional connectivity degree (FCD). Functional connectivity is estimated by Pearson linear correlation computed between every possible pair of voxels. The FCD is the count of total number of connections of a given voxel above a predefined threshold (Pearson linear correlation, p < 0.001). The FCD was calculated for each voxel at temporal windows of 7.8 s, 13.2 s, and 18.4 s with a step size of 1 sample for all the TRs. These windows were chosen since they were multiples of the measured TR in all experiments and, thus, consistent across all TR’s despite having a different number of samples. In addition, we performed the FCD calculation at a window of 26.4 s for the TR of 2.64 s to also evaluate the limit of the gain in FCD for this long TR measurement.

For each window size, the Pearson correlation was computed between the BOLD timecourses of each voxel corresponding to that respective window. The sliding windows were centered in time, i.e. we shifted the FCD time course by half the window size ahead in time.

The FCD time course from each region (functional ROI or regions from the 7-network atlas) was normalized by the average of all voxels within the gray matter mask. The hemodynamic response was also estimated in a similar way. To estimate the percent change in FCD (for the control task), the FCD values were extracted from the functional ROI (10 s before until 20 s after stimulus onset) and baseline corrected by subtracting the mean of the FCD values, 5 s prior to the stimulus onset. Similarly, the average FCD time course where extracted from regions defined by the 7-network parcellated atlas [24]. This atlas consists of seven main networks: visual, somatomotor, dorsal attention, ventral attention, limbic, frontoparietal and the default mode network. A region**-**wise baseline correction was performed by subtracting the mean of the FCD values 5 s prior to the stimulus onset. A 4 x 3 (TRs x Windows) within subject repeated-measures ANOVA was performed, using IBM® SPSS® statistics 22.0, with TRs (2.64 s, 1.32 s, 0.66 s and 0.33 s) and windows (7.8 s, 13.2 s and 18.4 s) to test the significance of FCD estimation in terms of peak amplitude (maximum change of the average FCD) and its timing i.e. timepoint of the peaks, across different approaches. The significance level was set to p < 0.05 (Greenhouse-Geisser corrected in case of non-sphericity) for each statistical analysis. Based on the preceding analysis we performed a paired t-test, which were Bonferroni-corrected for multiple comparisons. After Bonferroni correction, the threshold for a significant result was set to p = 0.05/30, approximately 0.001.

#### Dynamic FCD analysis in the patients with epilepsy

The dynamic FCD (dFCD) analysis in epilepsy patients was performed as for the control task using a window length of 13.2 s (20 samples) based on the results of the control experiments. IEDs were visually identified by an experienced rater on artifact-corrected EEG and IED onset times were noted. The FCD time course was extracted for a time window of 20 s before and 30 s after the IED onset from regions defined by the Freesurfer Desikan-Killiany anatomical atlas. In the case of patients, the percent change in FCD was computed with respect to the global mean, since we did not have external control on the occurrence of spikes/IEDs.

## Results

### Control task–ROI definition

**Fig 1:**
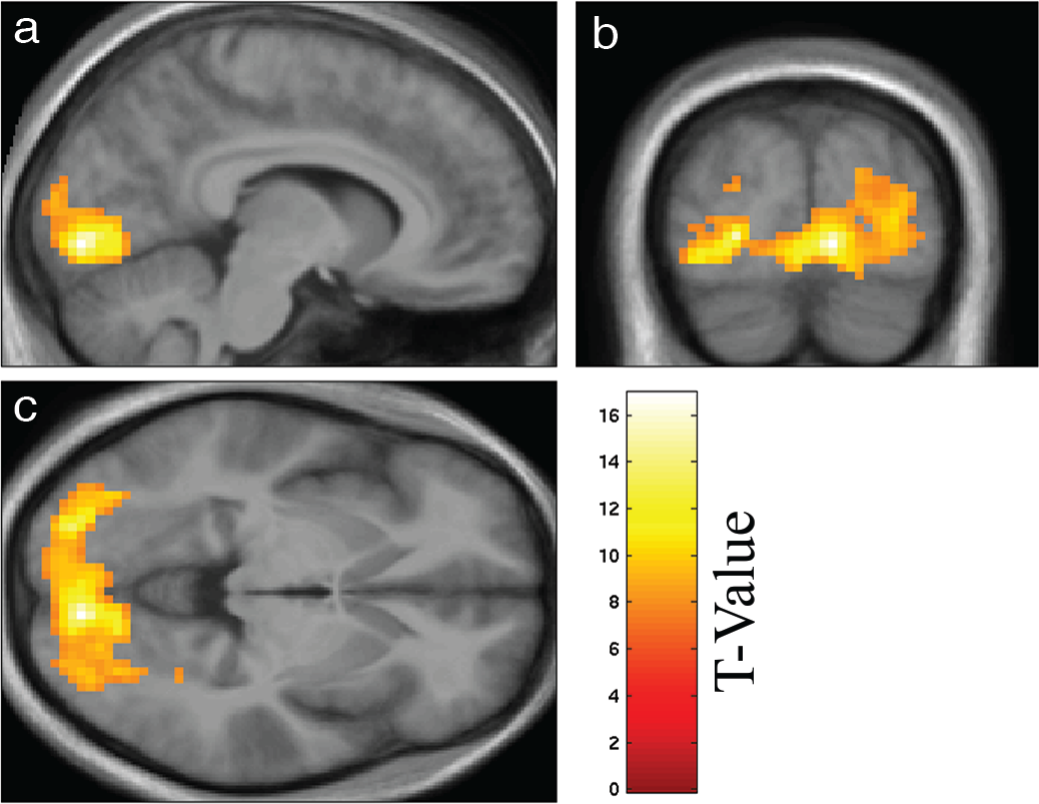
Activation during the block-design fMRI localizer. Region of interest as defined by the block-design with a height threshold of p < 0.05 (FWE corrected) displayed on sagittal (a), coronal (b), and transversal (c) slices of the mean normalized brain of the study participants.

As expected, the visual block-design (checkerboard > fixation cross) yielded widespread activation in the visual cortex (t (14) = 16.9, k = 1255 voxels, MNI coordinates: x = 9, y = -85, z = -5, see Fig 1, [17]). Participants successfully responded to 95.6 ± 1.4% (mean ± standard error) of the events, indicating good vigilance.

### FCD dynamics

**Fig 2:**
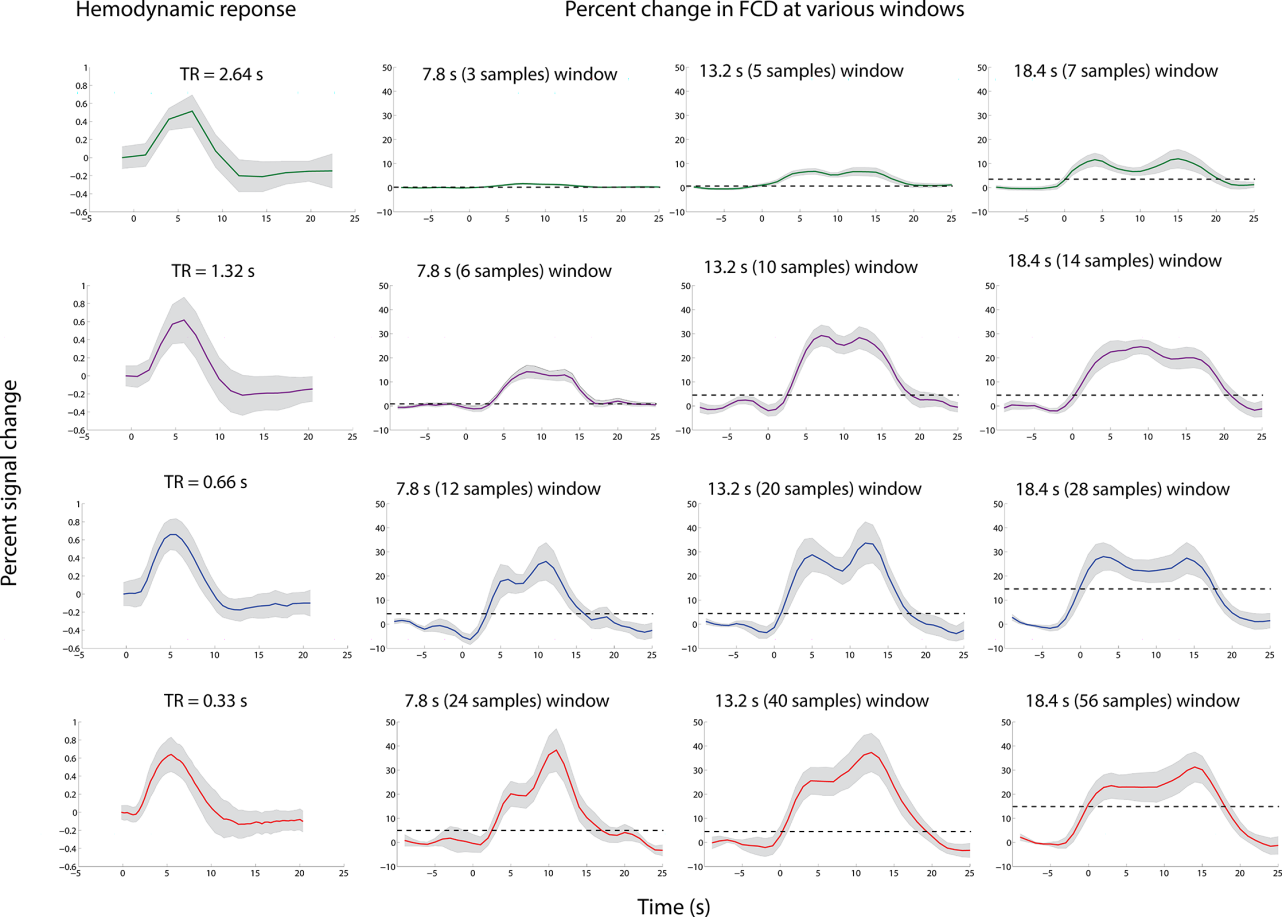
Comparison between the FCD percent change and the hemodynamic response. Percent change in FCD with stimulation beginning at 0 s for TR of 2.64 s (in green), 1.32 s (in purple), 0.66 s (in blue) and 0.33 s (in red) at various window sizes, along with the hemodynamic response. All these measures were computed in the region defined by the localizer. The 0 s indicates the stimulus onset. The horizontal dash line is a marker for significance (twice the mean standard error of the baseline).

We first compared the event-related hemodynamic response (Fig 2, first column) with the FCD transients (Fig 2, columns 2–5 for window sizes of 7.8 s, 13.2 s, and 18.4 s respectively) for the TRs of 2.64 s, 1.32 s, 0.66 s, and 0.33 s within the defined ROI obtained from the control task. For short TRs at a window size of 7.8 s and 13.2 s, FCD in the ROI peaked around 5–6 s after stimulus onset, which follows the behavior of the event-related BOLD timecourse. Interestingly, a second peak around 12–13 s after stimulus onset was also observed. Although increasing the window size resulted in some temporal smoothing of the FCD time course, these two peaks were always reliably identified.

### Effect of TR and window size

The two-way ANOVA revealed a significant main effect for the factor TR for the amplitude of both peak 1 (F (1.997, 27.96) = 11.335, p < 0.001) and peak 2 (F (2.005, 28.072) = 11.581, p < 0.001). The long TR of 2.64 s always resulted in lower FCD values as compared to the shorter TRs (1.32 s, 0.66 s, and 0.33 s). For both peaks, this effect was significant (p < 0.001) for the window sizes of 7.8 s and 13.2 s. For the window size of 18.4 s, this effect scarcely failed to reach significance for peak 2 when comparing the TRs of 2.64 s and 0.66 s and for both peaks when comparing the TRs of 2.64 s and 0.33 s (see S1 Table).

**Fig 3:**
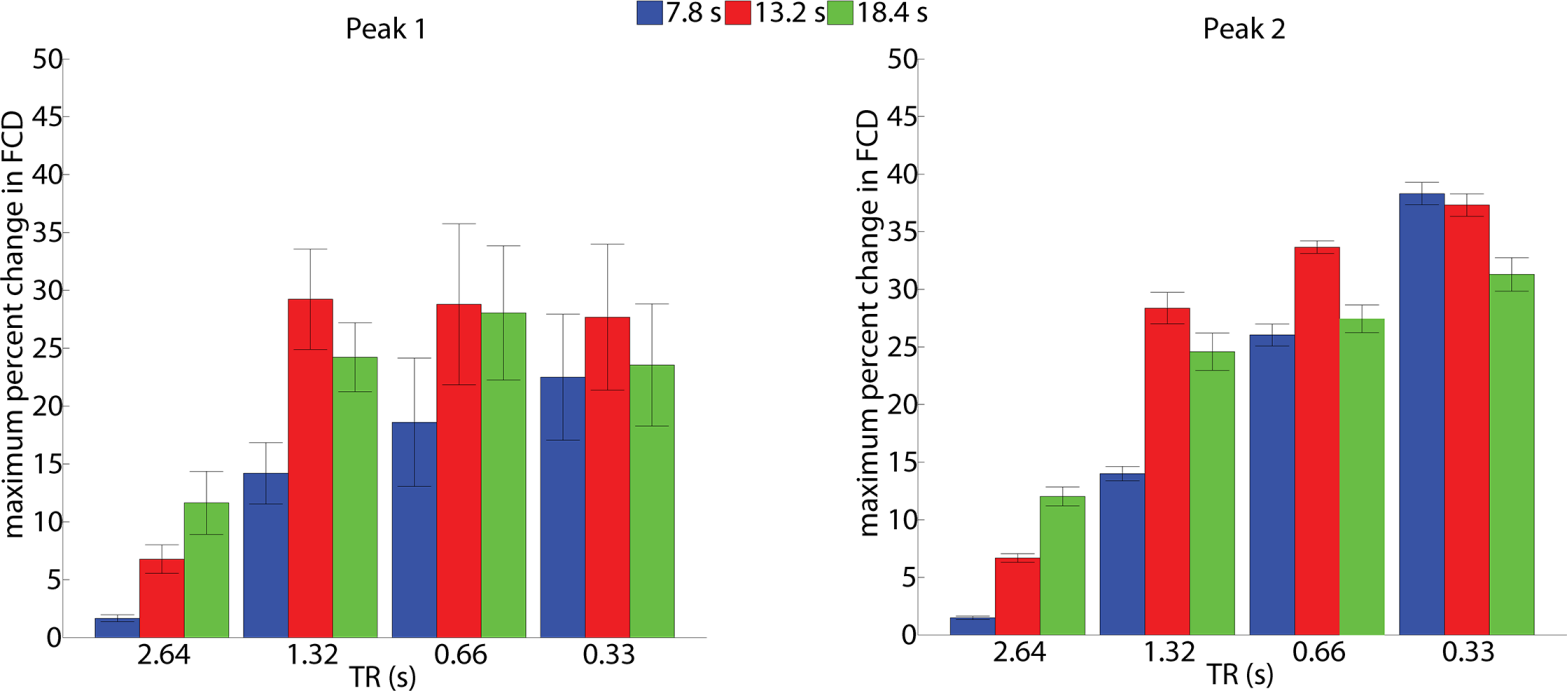
Maximum percent change in FCD across TRs and window sizes. Maximum percent change of FCD during the event-related task in the ROI, for peak 1 and peak 2 across window sizes of 7.8 s, 13.2 s and 18.4 s at TRs ranging from 2.64 s to 0.33 s.

We also observed a significant main effect on FCD amplitude for the factor window length for both peak 1 (F (1.293, 18.101) = 18.743, p < 0.001) and peak 2 (F (1.44, 20.272) = 6.183, p = 0.013). For the TR of 2.64 s the FCD increased approximately linearly with window length (Fig 3). Accordingly, the post-hoc paired t-tests revealed significantly (p < 0.001) higher FCD with increasing window lengths for both peaks. For peak 2, however, this effect only showed a strong trend towards significance (p = 0.0079), which failed to remain significant after Bonferroni correction. At the TR of 1.32 s, a significant increase of the FCD change was observed when comparing the window size of 7.8 s with the window sizes of 13.2 s and 18.4s, but not for the comparison of the window sizes of 13.2 s and 18.4 s. At the short TRs of 0.66 s and 0.33 s no significant effects of window size on FCD changes were observed.

Finally, the interaction of window length and TR for both peak 1 (F (2.740, 38.355) = 4.927, p = 0.007) and peak 2 (F (2.647, 37.053) = 7.293, p = 0.001) was also significant. FCD percent change for both the peaks showed an approximately linear increase with increasing window sizes for the long TR of 2.64 s. For the short TRs the FCD percent change showed saturation after an initial increase from window size 7.8 s to 13.2 s with an inverted u-shape behavior.

In terms of the time point of the two peaks, the three-way ANOVA revealed a significant effect of the window size (18.4 s) for peak 2 (F (1.379, 19.313) = 14.869, p = 0.001) whereas the effect for peak 1 was at trend level (F (1.667, 23.342) = 4.020, p = 0.038). No effect of TR was found (all p>0.2) for the time point of both peaks.

### Spatial distribution of FCD (control task)

**Fig 4:**
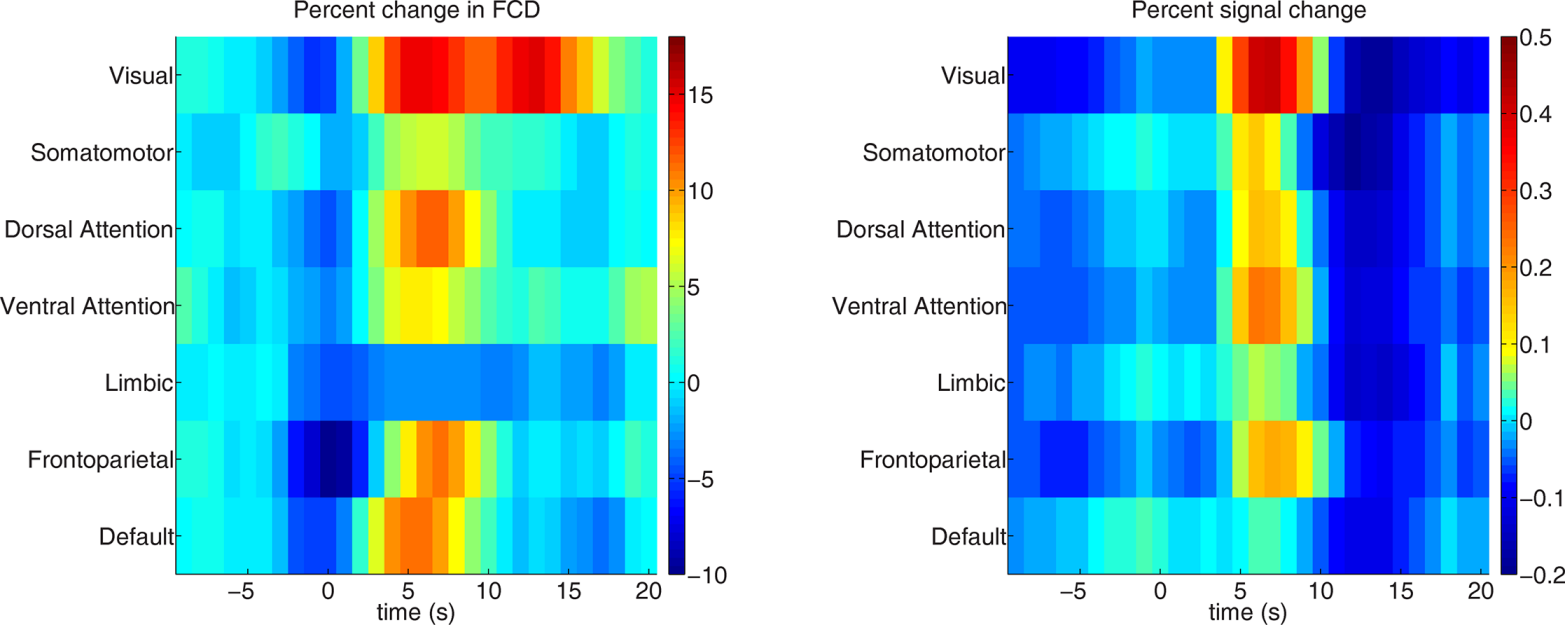
Mean FCD percent change and percent signal change across atlas based regions. Mean FCD percent change along with the percent signal change across time (-10 to 20 s) for functional regions as defined by the [24] atlas at window size of 13.2 s (TR = 0.66 s). The 0 s indicates the stimulus onset.

Fig 4 shows the spatial and temporal distribution of the FCD during the event-related paradigm across the regions defined by the functional network atlas [24]. In addition, it also shows the percent signal change (hemodynamic response) across the functional regions. As expected, the highest percent change in FCD (30% increase) was observed 5–6 s after stimulus onset in the ROI defined by the block design (Fig 2). We also observed widespread increase in FCD across various functionally defined regions. The visual area that is partly overlapping with the functional ROI showed an 18% increase in FCD relative to stimulus onset. Regions which are associated to the dorsal attention and frontoparietal network also showed a 10% increase in FCD after stimulus onset. In addition, we also observed an increase in FCD percent change in regions which are assigned to the default mode network. In contrast to this, the raw BOLD signal in the default mode network was negative, whereas other regions (including the visual and attention areas) showed a BOLD signal increase.

### Spatial distribution of FCD (patients)

**Fig 5:**
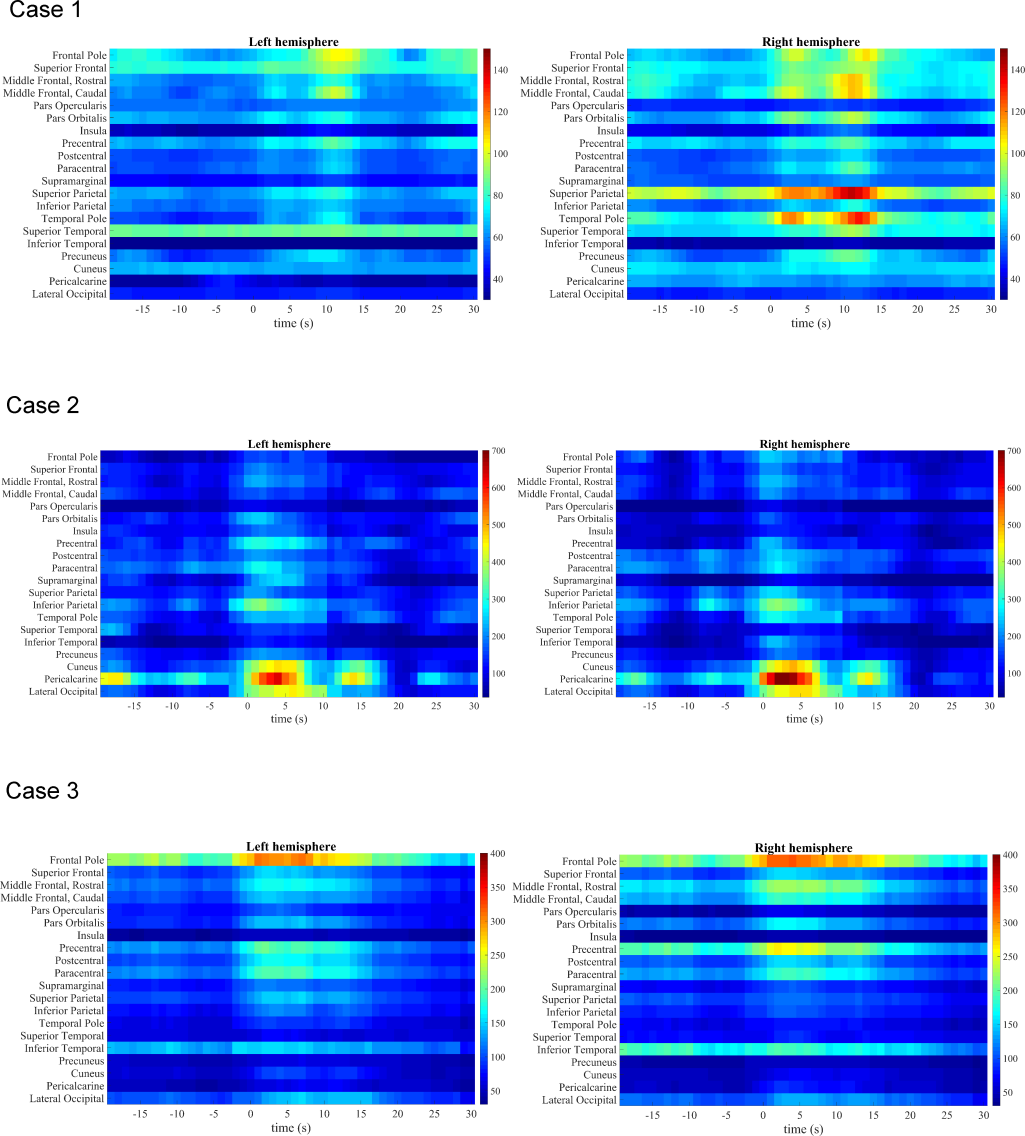
Mean FCD across atlas based regions in epileptic patients. Mean FCD across time (-20 to 30 s) for cortical regions as defined by the Freesurfer atlas. The 0 s indicates the IED onset.

Since the maximum value of peak 1 was observed at window size of 13.2 s for all sub-second TR’s, we chose a window of 13.2 s for the subsequent patient data analysis. Patient 1 (case 1, Fig 5) had an epileptogenic, low-grade tumor near the right posterior hippocampus/mesio-parietooccipital region. The FCD map also showed the strongest increase in FCD in parietal regions but also, with lower values, in frontal regions both with clear right lateralization. The onset of the FCD increase was slightly after (0.9 s later) the IED appearance in the EEG (150% increase in the right superior parietal region). The conventional EEG-fMRI showed right-lateralized insular activations (S2 Fig), clearly more anterior than the FCD maps (Fig 5 and S3 Fig). These were also visible in the raw BOLD timecourse (S4 Fig), however, no obvious parietal BOLD increase or decrease was noted. Patient 2 (case 2, Fig 5) had idiopathic/genetic generalized epilepsy (IGE/GGE) with generalized IED. The dFCD map showed a bilateral increase (600%) in dFCD (especially in occipital and parietal regions) that occurred slightly before (2.3 s prior) the IED onset. The conventional EEG-fMRI analysis showed thalamic activations and deactivations with parieto-occipital focus (S2 Fig). The raw BOLD timecourse was unrevealing at the onset and only showed cuneal/pericalcarine decrease around 15 seconds after the onset (S4 Fig). Patient 3 (case 3, Fig 5) is another IGE/GGE patient that had generalized IEDs. This patient also showed a strong bilateral increase (300%) of FCD, in this case in frontal regions, slightly before (2.3 s prior) the IED onset. The conventional EEG-fMRI showed bilateral mesiofrontal and insular/opercular activations with deactivation parieto-occipital. The raw BOLD timecourse confirmed parieto-occipital signal decrease 10–15 seconds after the IED but no clear signal change at the onset (S4 Fig).

## Discussion

In this study, we examined the impact of TR and window size on dynamic functional connectivity degree (dFCD) in a sliding window approach in event-based paradigms. We found plausible network changes in a simple visual control task. In addition, we also applied the dFCD in three epilepsy patients with spontaneous IEDs and observed FCD increases related to IED onset.

One of the most striking finding was that it was possible to capture dynamic network changes using BOLD-fMRI and the dFCD approach for brief events of 500 ms even with a relatively short window size of 7.8 s. Our control task, a pseudo-random visual stimulation paradigm based on real IED patterns, involved a strong visual component (checkerboard stimulation) but also a motor component (button press) and attention shift. Hence, we expected to observe FCD changes in the visual areas but also some involvement of the somatomotor, the attention and frontoparietal and default mode networks. The global maximum of FCD was invariably in the primary visual networks (both localizer- as well as atlas-based). As this increase in FCD is 5–6 s relative to the stimulus onset we consider them as neural responses due to the task. However, FCD analyses cannot distinguish between FCD increases due to activations or deactivations of BOLD responses. Both will result in positive correlations (and thus positive FCD changes) if these occur in several voxels in synchrony. This can be observed in the default-mode network, which is a classical “task-negative” area. Yet the FCD values also increased in this network, whereas the BOLD response was clearly negative, as expected. This is a conceptual issue when interpreting all correlation-based FC methods.

We found a double-peak pattern of FCD transients in the control task, which consistently occurred in all applied analyses. The origin of this phenomenon is not clear so far. The observation that this double-peak pattern was restricted to the visual cortex and the fact that they roughly occur at the onsets of the peak maximum and of the undershoot of the event-related BOLD response suggest a physiologic origin. It should be noted, however, that the time between the two peaks increased with applied window size indicating that this phenomenon is sensitive to analysis parameters. We verified this by separate 2-way ANOVAs on the peak onsets. Indeed, this revealed a significant interaction between TR and window size for the onset of peak 2 (and scarcely failed to reach significance for peak 1).

Further studies are needed to fully disambiguate physiologic factors and potential influences of analysis parameters driving the observed double-peak phenomenon.

We found significant FCD amplitude differences between the conventional TR of 2.64 s and the shorter TRs provided by the MB accelerated EPI. Using a conventional TR of 2.64 s only marginal FCD increases were detectable, e.g. at a window of 13.2 s a 6% increase in FCD compared to a 28% increase for a TR of 0.66 s at the same window length. This highlights that conventional EPI is not well suited to study dFC of brief events. Faster sampling is clearly advisable, in our experiments TR = 1.32 s (MB = 2), TR = 0.66 s (MB = 4) and TR = 0.33 s (MB = 8) could achieve almost equivalent FCD values using the longer window sizes (13.3 s and above). In terms of the window size, we found an inverted u-shaped behavior for both peaks. This is expected since longer windows would improve the stability of the FCD estimate due to more data points, but at the cost of increased temporal smoothness, thereby decreasing the FCD estimates of events that are much shorter than the window lengths. Therefore, we suggest determining the “ideal” window size based on experimental and technical factors. In our case for peak 1, 18.4 s window size for TR = 2.64 s gave the maximal FCD amplitude and 13.2 s for the other TRs. This was identical for peak 2 with the exception of TR = 0.33s; here the maximum was observed at the 7.8 s window.

Most studies on dynamic functional connectivity have been carried out in resting-state; event-related designs have, so far, been less well studied [29]. In addition, these few studies have been carried out based on a-priori chosen seeds or regions, for which time-varying correlation coefficients have been estimated [9, 29]. Whereas here we have evaluated dFCD of the whole brain, i.e. all voxels within a gray matter mask. The use of short windows can be relevant even for block paradigms, where the task period is often in the order of 10–30 s, to observe dynamic change during the task [29]. Since the BOLD signal is relatively slow compared to the true neuronal activity, one may argue that such short windows may not be necessary to evaluate dFC. However, emerging evidence suggests that FC may be present in components of the fMRI signal above 0.1 Hz [30]. Here we showed that maximum percent change in FCD for the data obtained at TRs of 1.32 s, 0.66 s and 0.33 s were significantly higher when compared with the conventional TR of 2.64 s. These results indicate that conventional TRs of around 2–3 s are not well suited to estimate FCD effectively for brief events. Although our study was done with an event-based approach, it is likely that TR and window size will have a relevant effect on resting-state dFC estimates as well. Hindriks et al. 2016 could not detect significant dynamic FC in resting state during 5–10 min scan duration sampled at TR = 2 s with a window length of 2 minutes [9]. Additionally, the time course of the dFCD at short windows (≤ 13.2 s) shows that, this approach is capable of capturing functional connectivity across various regions of the brain. However, further studies are needed to assess the effect of sampling variability versus true connectivity especially for resting-state studies. Using the optimized FCD estimation parameters from the control task (TR = 0.66 s, window size = 13.2 s) we demonstrated dynamic FCD changes in epilepsy patients related to the IED in simultaneous EEG-fMRI. We showed the feasibility of this approach by detecting transient activity in the three patients (one focal lesional, two with generalized epilepsy). Interestingly the onset of FCD was slightly before the IED appearance for both IGE/GGE patients, whereas it was slightly after the IED onset for the focal patient. Early BOLD responses could be related to epileptic activity not detected on scalp EEG [31]. There were divergences between conventional EEG-fMRI / BOLD timecourse and the dFCD analysis. Particularly, the FCD changes occurred earlier, had partially different topology and much higher signal amplitude. However, further studies with larger patient cohorts are needed to draw clinical conclusions, validate the differences to conventional EEG-fMRI or make a neurobiological distinction of different epilepsy syndromes. Still, these results indicate a potential of dFCD to study the temporal characteristics of IED and, potentially, seizure generation.

### Limitations

One of the major concerns in a sliding window analysis is the issue of noise, which could be technical (scanner related), physiological (due to the cardiovascular system) or neuronal noise. Variations in the magnitude of noise levels across the scan, as well as non-neuronal events that generate strong spatially correlated signal fluctuations could influence the FCD estimation. While there are a number of techniques for reducing noise (e.g. RETROICOR, RVHRCOR, CompCor [32, 33]), residuals unavoidably remain, therefore a better understanding of how to remove noise from fMRI time series is critical. Such generalized physiological correction methods could even introduce spurious connections [34]. However, the impact of physiological noise was minimal in cortical structures in our event-related design, as shown in our previous work [17]. Head motion also could have adverse effect on dynamic connectivity analysis. These movements can introduce strong signal fluctuations that can manifest as temporary changes in connectivity patterns [18]. The generation of an appropriate null distribution is necessary to perform statistical testing in dFCD studies. There are resting-states studies which have used a sliding-window approach to quantify the time varying behavior of the fMRI using appropriate statistical testing in real as well as simulated surrogate data [9, 35]. These methods could be applied to quantify the dFCD time course in event-related designs; however, this is beyond the scope of the present study.

## Conclusion

MB EPI is an important MRI technique capable of providing improved temporal resolution, which can be used to facilitate dynamic connectivity analysis. In fact, in our study dFCD transients were only marginally detectable with conventional TR fMRI, whereas sub-second MB EPI revealed consistent FCD changes. In our paradigm of brief events, a window size of 7.8–13.2 s (sampled beyond the conventional TR of 2.64 s) would be an optimum choice. With this approach, it was possible to capture widespread network changes across various functional regions for brief visual stimulation. In addition, we could show the potential feasibility of the dFCD approach to detect transients during epileptic spike discharges. A larger cohort of patients is required to assess the stability and clinical utility of this method in epilepsy. We argue that the optimization of FCD processing parameters is relevant for a broad variety of event-related paradigms and needs to be considered when planning and interpreting such studies. Such approaches could also be used in various brain diseases, which are characterized by transient alterations of brain function.

## Supporting information

S1 Fig Maximum percent change in FCD across windows for TR of 2.64 s. Maximum percent change of FCD during the event-related task in the ROI, for peak 1 and peak 2 across window sizes of 7.8 s, 13.2 s, 18.4 s and 26.4 s at a TR of 2.64 s.
(TIF)

S2 Fig Standard first level SPM results at FWE corrected (P < 0.05). The results have been overlaid for activation and deactivation on the subject specific T1 normalized image.
(TIF)

S3 Fig FCD maps at time point zero (onset of IED). FCD values overlaid on the subject specific T1 normalized image.
(TIF)

S4 Fig Mean BOLD signal across atlas based regions in epileptic patients. Mean BOLD signal across time (-20 to 30 s) for cortical regions as defined by the Freesurfer atlas. The 0 s indicates the IED onset.
(TIF)

S1 Table Paired t-test across windows and TRs (p < 0.05, Bonferroni corrected) for peak 1 and peak 2. (DOCX)
