# Correction: Evaluating the impact of fast-fMRI on dynamic functional connectivity in an event-based paradigm

**DOI:** 10.1371/journal.pone.0195916

**Published:** 2018-04-11

**Authors:** Ashish Kaul Sahib, Michael Erb, Justus Marquetand, Pascal Martin, Adham Elshahabi, Silke Klamer, Serge Vulliemoz, Klaus Scheffler, Thomas Ethofer, Niels K. Focke

There are errors in the graphs for the 18.4 s window in Fig 2. Please see the correct Fig 2 here.

**Fig 2:**
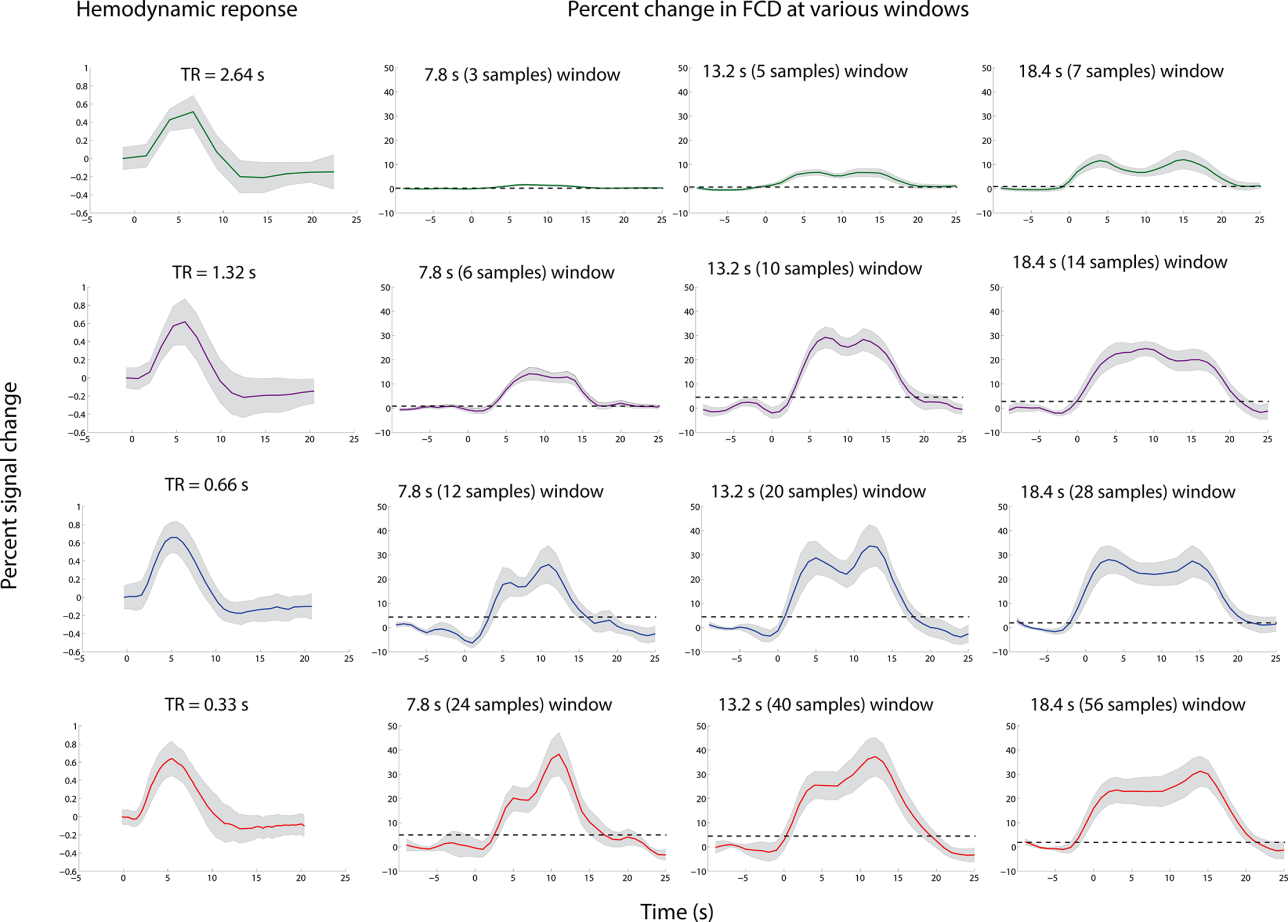
Comparison between the FCD percent change and the hemodynamic response. Percent change in FCD with stimulation beginning at 0 s for TR of 2.64 s (in green), 1.32 s (in purple), 0.66 s (in blue) and 0.33 s (in red) at various window sizes, along with the hemodynamic response. All these measures were computed in the region defined by the localizer. The 0 s indicates the stimulus onset. The horizontal dash line is a marker for significance (twice the mean standard error of the baseline).
