# Supplementary figures and images for: Evaluating the impact of fast-fMRI on dynamic functional connectivity in an event-based paradigm
Source: PLoS One. 2018 Jan 22;13(1):e0190480. doi: 10.1371/journal.pone.0190480 (PMC5777653; doi:10.1371/journal.pone.0190480)

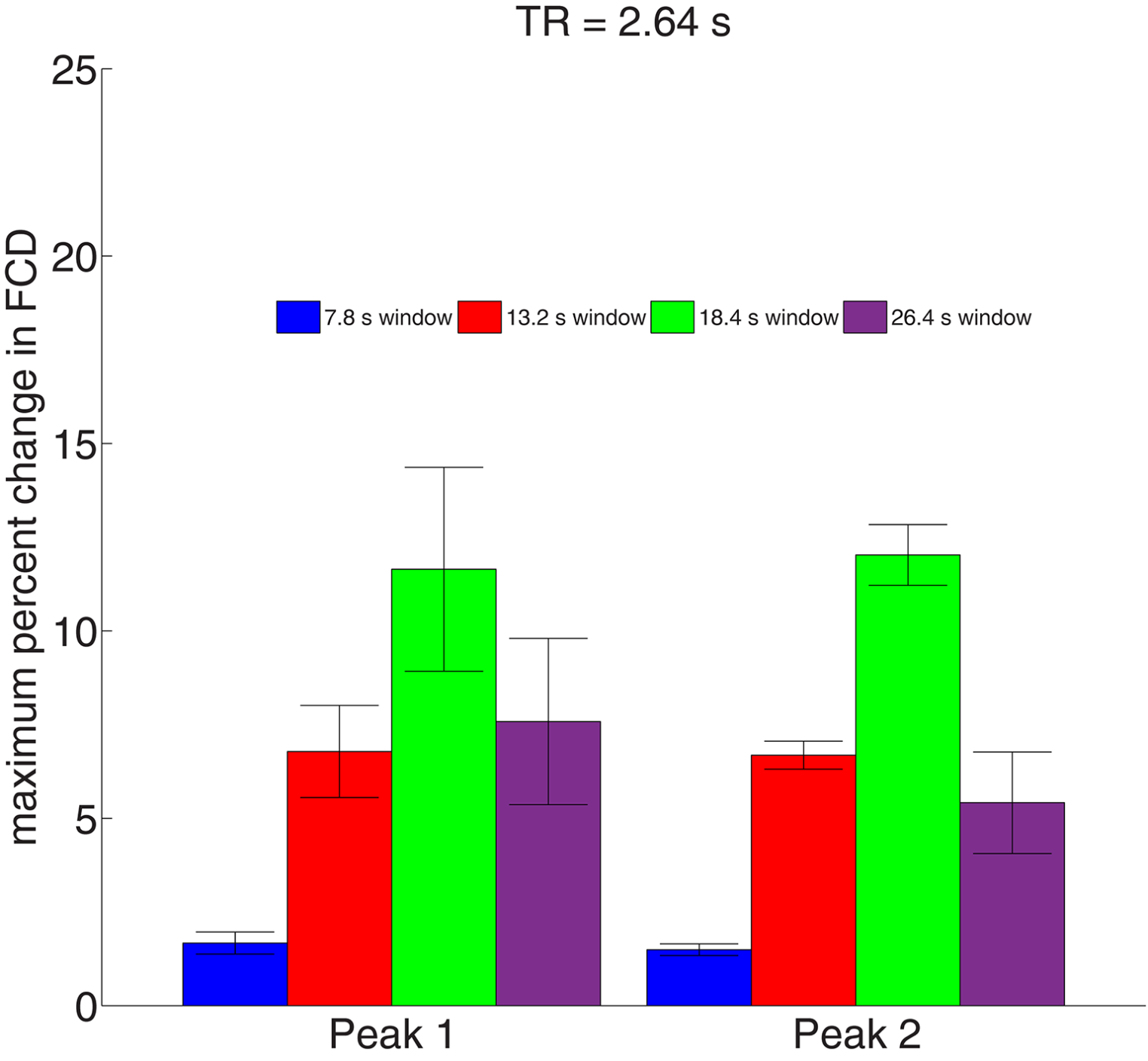

Supplement: S1 Fig — Maximum percent change of FCD during the event-related task in the ROI, for peak 1 and peak 2 across window sizes of 7.8 s, 13.2 s, 18.4 s and 26.4 s at a TR of 2.64 s. (TIF) [file pone.0190480.s001.tif]

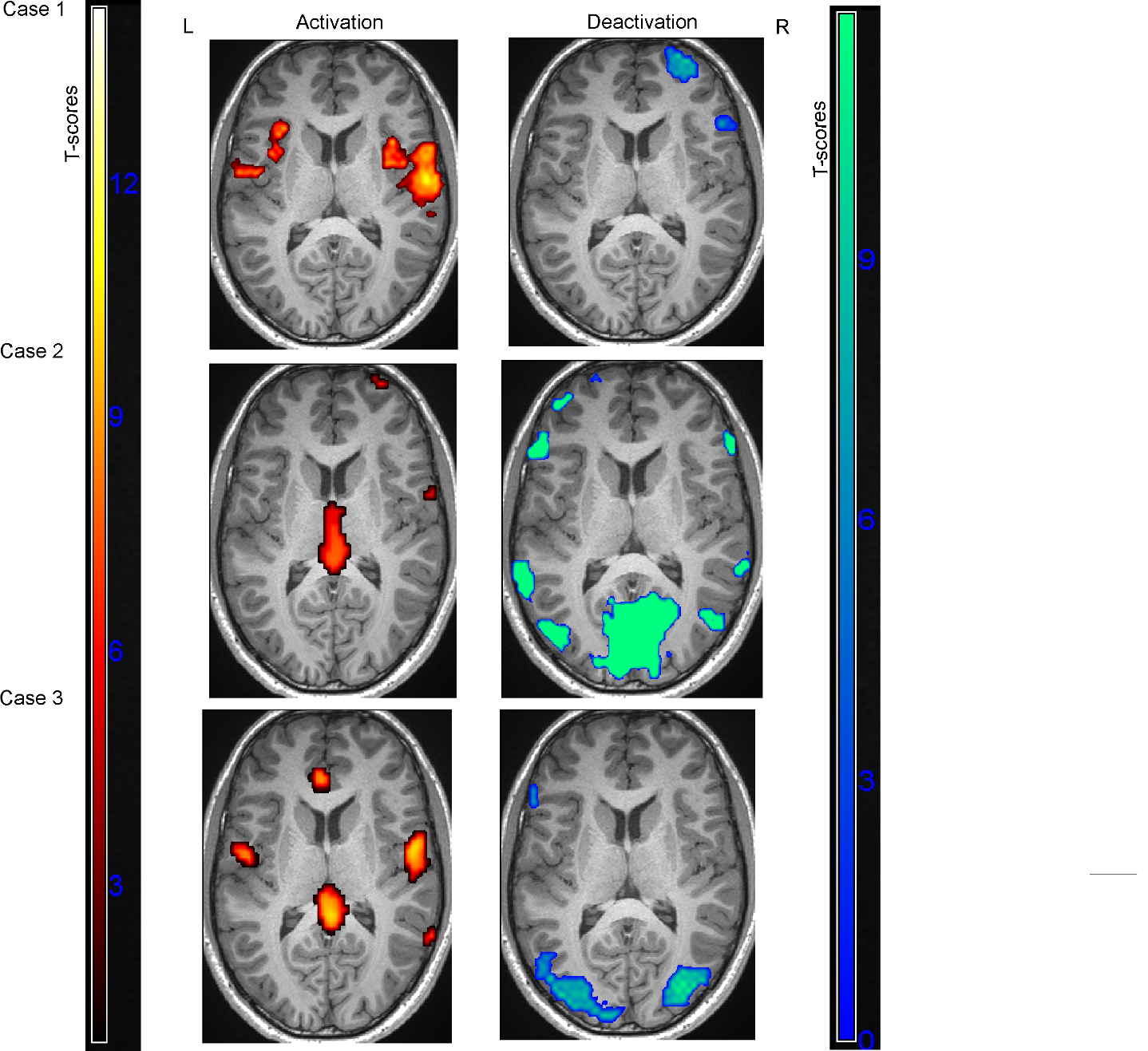

Supplement: S2 Fig — The results have been overlaid for activation and deactivation on the subject specific T1 normalized image. (TIF) [file pone.0190480.s002.tif]

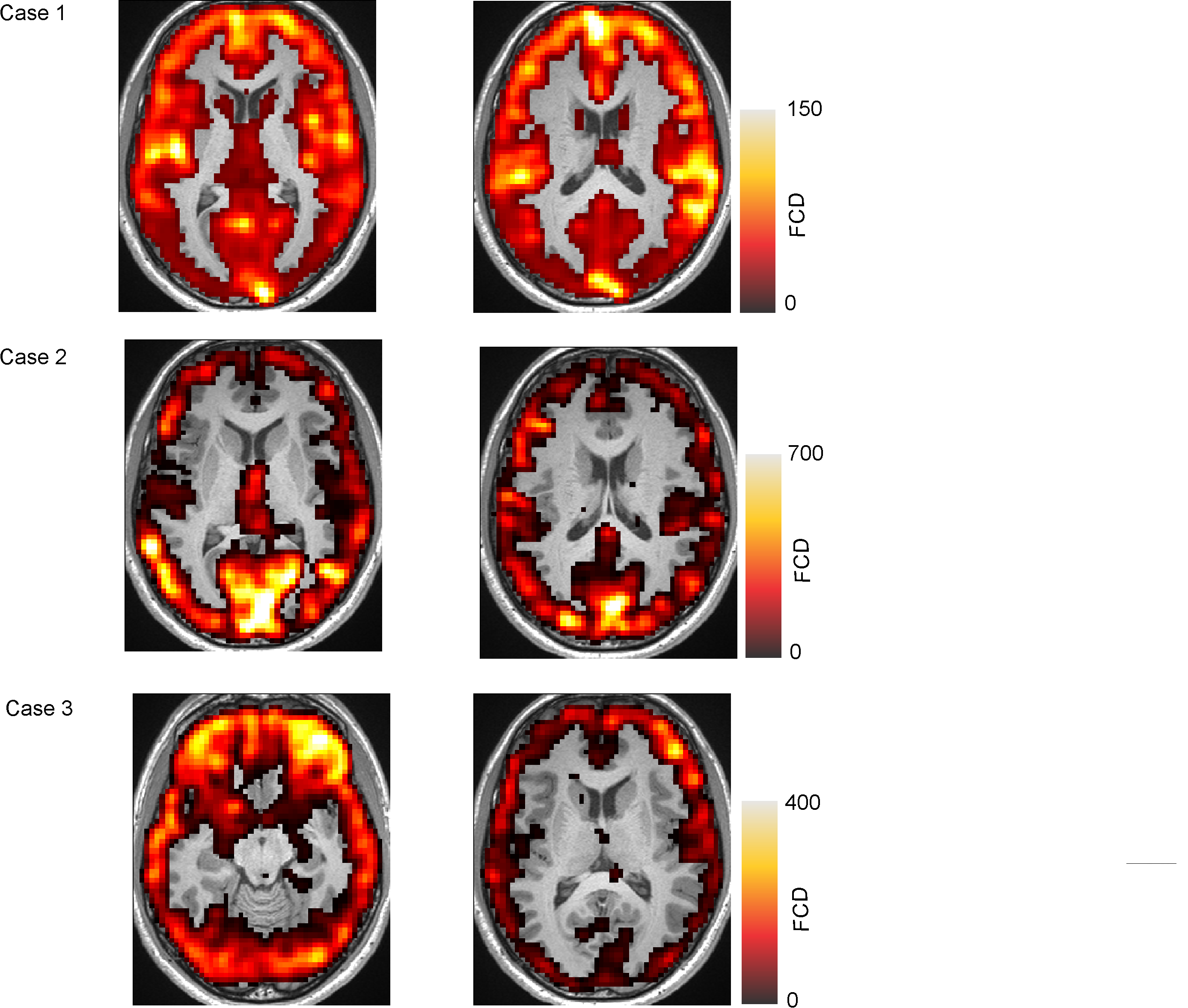

Supplement: S3 Fig — FCD values overlaid on the subject specific T1 normalized image. (TIF) [file pone.0190480.s003.tif]

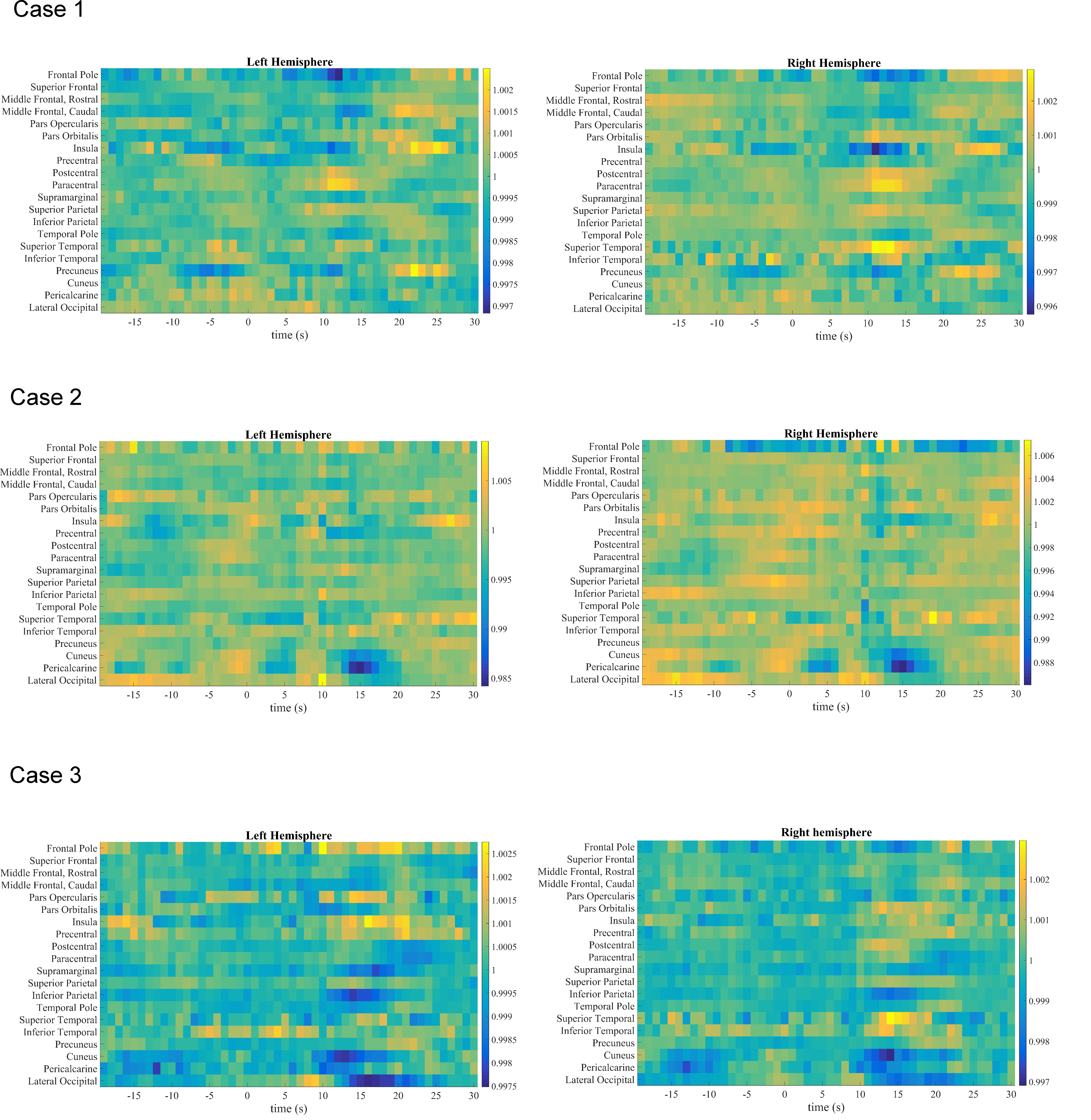

Supplement: S4 Fig — Mean BOLD signal across time (-20 to 30 s) for cortical regions as defined by the Freesurfer atlas. The 0 s indicates the IED onset. (TIF) [file pone.0190480.s004.tif]
