# Supplementary material for: Evaluating the impact of fast-fMRI on dynamic functional connectivity in an event-based paradigm
Source: PLoS One. 2018 Jan 22;13(1):e0190480. doi: 10.1371/journal.pone.0190480 (PMC5777653; doi:10.1371/journal.pone.0190480)
Supplement: S1 Table — (DOCX) [file pone.0190480.s005.docx]

**S1 Table. Paired t-test across windows and TRs (p < 0.05, Bonferroni corrected) for peak 1 and peak 2.**

| **TR (s), window (s)** | **TR (s), window (s)** | **p (peak 1)** | **p (peak 2)** |
| --- | --- | --- | --- |
| 2.64, 7.8 | 1.32, 7.8 | **< 0.001** | **< 0.001** |
| 2.64, 7.8 | 0.66, 7.8 | **< 0.001** | **< 0.001** |
| 2.64, 7.8 | 0.33, 7.8 | **< 0.001** | **< 0.001** |
| 1.32, 7.8 | 0.66, 7.8 | 0.021 | 0.0182 |
| 1.32, 7.8 | 0.33, 7.8 | 0.0046 | 0.0015 |
| 0.66, 7.8 | 0.33, 7.8 | 0.238 | 0.0247 |
| 2.64, 13.2 | 1.32, 13.2 | **< 0.001** | **< 0.001** |
| 2.64, 13.2 | 0.66, 13.2 | **< 0.001** | **< 0.001** |
| 2.64, 13.2 | 0.33, 13.2 | **< 0.001** | **< 0.001** |
| 1.32, 13.2 | 0.66, 13.2 | **< 0.001** | 0.3547 |
| 1.32, 13.2 | 0.33, 13.2 | 0.278 | 0.1616 |
| 0.66, 13.2 | 0.33, 13.2 | 0.4435 | 0.2742 |
| 2.64, 18.4 | 1.32, 18.4 | **< 0.001** | **< 0.001** |
| 2.64, 18.4 | 0.66, 18.4 | **< 0.001** | 0.002 |
| 2.64, 18.4 | 0.33, 18.4 | 0.0038 | 0.0055 |
| 1.32, 18.4 | 0.66, 18.4 | 0.1409 | 0.3443 |
| 1.32, 18.4 | 0.33, 18.4 | 0.2888 | 0.2093 |
| 0.66, 18.4 | 0.33, 18.4 | 0.2282 | 0.3409 |
| 2.64, 7.8 | 2.64, 13.2 | **< 0.001** | **< 0.001** |
| 2.64, 7.8 | 2.64, 18.4 | **< 0.001** | **< 0.001** |
| 2.64, 13.2 | 2.64, 18.4 | **< 0.001** | 0.0079 |
| 1.32, 7.8 | 1.32, 13.2 | **< 0.001** | **< 0.001** |
| 1.32, 7.8 | 1.32, 18.4 | **< 0.001** | **< 0.001** |
| 1.32, 13.2 | 1.32, 18.4 | 0.0311 | 0.0421 |
| 0.66, 7.8 | 0.66, 13.2 | 0.0017 | 0.0254 |
| 0.66, 7.8 | 0.66, 18.4 | 0.0043 | 0.4862 |
| 0.66, 13.2 | 0.66, 18.4 | 0.3451 | 0.0451 |
| 0.33, 7.8 | 0.33, 13.2 | 0.0763 | 0.3809 |
| 0.33, 7.8 | 0.33, 18.4 | 0.4491 | 0.0738 |
| 0.33, 13.2 | 0.33, 18.4 | 0.0044 | 0.0056 |
